# Functional abdominal pain causing Scurvy, Pellagra, and Hypovitaminosis A

**DOI:** 10.12688/f1000research.3-35.v1

**Published:** 2014-02-04

**Authors:** Edith Y. Ho, Christian Mathy

**Affiliations:** 1 Division of Gastroenterology, Department of Medicine, Case Western Reserve University / Louis Stokes Cleveland Veterans Affairs Medical Center, Cleveland, OH 44106, USA; 2 Division of Gastroenterology, Department of Medicine, University of California, San Francisco, CA 94143, USA

## Abstract

Severe vitamin deficiency disease is rarely seen in developed countries. We present an atypical case of a young man with scurvy, pellagra, and hypovitaminosis A, caused by longstanding functional abdominal pain that severely limited his ability to eat.

## Case presentation

This case describes a spirited 20 year-old male Caucasian college student, with no significant past medical history, who has been suffering from severe mid-epigastric and lower abdominal pain for ten years. He has seen multiple doctors for these symptoms and was diagnosed with irritable bowel syndrome. Various medical therapies were tried but they did not provide durable benefit. He described having acute onset of sharp, debilitating pain, lasting twenty minutes to several hours on a daily basis. The pain was often triggered by eating or drinking. Associated gastrointestinal symptoms included watery diarrhea. Over the last five years, the pain had become so severe, especially post-prandially, that he could only tolerate small amount of plain food, such as plain white bread and red meat, and drink only water. As a result, he had difficulty gaining weight and was frustrated with his dietary limitations. He also complained of blurry vision at night. Upon physical examination, the patient appeared thin but not cachectic. His examination was notable for dermatitis of his dorsal hands bilaterally demarcating at the wrists with hyperkeratosis over his metacarpophalangeal and proximal interphalangeal joints. Follicular hyperkeratosis with perifollicular hemorrhage and corkscrew hair were apparent on his thighs, calves, and buttocks symmetrically.

The patient underwent an extensive workup for chronic abdominal pain. His initial laboratory tests, including a complete blood count, metabolic panel, liver profile tests, and fecal fat tests were unremarkable. He underwent an abdominal MRI, CT enterography, cholescintigraphy (HIDA scan), upper endoscopy, colonoscopy, capsule endoscopy, endoscopic ultrasound and a gastric emptying study, which were all unrevealing. He was tested as positive for *Helicobacter pylori* infection and small intestinal bacterial overgrowth, for which he received an appropriate course of antibiotics, but his symptoms persisted. Screenings for heavy metal exposure and porphyria were negative. When vitamin levels were checked, he was found to have undetectable levels of vitamin A and vitamin C. Clinical suspicion of severe nutritional deficiency was further confirmed by his dermatologist who commented that the patient’s pattern of photodistributed, erythematous patches, well demarcated on dorsal hand with hyperkeratosis” over his finger joints was consistent with pellagra and “follicular hyperkeratosis with perifollicular hemorrhage and corkscrew hair” were consistent with vitamin A and C deficiencies. Based on this patient’s clinical presentation, laboratory values, as well as dermatopathological findings, diagnoses of pellagra (vitamin B3 or niacin deficiency), scurvy (vitamin C deficiency), and phrynoderma (vitamin A deficiency) were made.

## Course follow-up and outcome

The patient was started on intravenous infusions of vitamin C and liquid multivitamin for niacin and vitamin A repletion. To treat his abdominal pain, the patient tried a number of pharmacological therapies, including antispasmodic agents, tricyclic antidepressants, and gabapentin, none of which provided relief. The patient was referred for psychiatry and to a pain specialist for further management. After a few months of nutritional supplementation, his vitamin levels returned to the normal range. His diarrhea and night vision also improved.

## Discussion

Vitamin deficiency diseases are common in developing countries where there is limited access to a healthy diet, particularly with fruits and vegetables. In industrialized countries, nutritional deficiencies are rarely seen, but can occur in alcoholics, institutionalized elderly, or those with restrictive dietary intake related to eating disorders, psychiatric conditions, food allergies, or organic gastrointestinal disorders ^1– 5^. This case highlights three specific vitamin deficiencies: scurvy, pellagra, and hypovitaminosis A.

Scurvy is a clinical syndrome that results from ascorbic acid or vitamin C deficiency, largely due to impaired collagen synthesis resulting in defective connective tissue. Symptoms can occur as early as three months after insufficient intake. Symptoms include ecchymosis, petechiae, coiled hairs, hyperkeratosis, bleeding gums, arthralgia, Sjogren’s syndrome, and poor wound healing. Scurvy generally occurs when the plasma concentration of ascorbic acid is < 0.2 mg/dL (11 µmol/L). Humans require exogenous intake of ascorbic acid for maintenance, particularly from fruit and vegetables. The recommended dietary allowance for ascorbic acid is 75 mg per day for most women, 90 mg per day for men, and 120 mg per day for pregnant/lactating women or the elderly ^6, 7^.

Niacin, or vitamin B3, deficiency can cause pellagra, a clinical syndrome classically referred to as the four D’s: diarrhea, dermatitis, dementia, and death. The most common finding is a symmetric hyperpigmented rash in exposed areas of skin, similar to a sunburn ^4^. The recommended dietary allowance for niacin is 14 NEs (Niacin Equivalents) daily for adult females, 16 NEs for adult males, 17 NEs for pregnant women, and 18 NEs for lactating mothers. One NE is equivalent to 1 mg of niacin, which is equal to 60 mg of dietary tryptophan ^7, 8^.

Vitamin A is essential to cellular differentiation and integrity of the eye. Therefore, deficiency may lead to eye dryness, fragility, and night blindness. It is also associated with impaired bone growth, hyperkeratosis, and poor immune function. The recommended daily allowance for vitamin A is 3000 IU (international units) (900 µg retinol) daily for adult males and 2300 IU (700 µg retinol) for females ^7^.

One important lesson to be gleaned from this case report is the potentially serious complications of a functional disorder. In this case, the patient suffered from severe chronic abdominal pain resulting in dietary restrictions that ultimately led him to profound malnutrition. If left untreated, this could progress to severe morbidity and even death in extreme cases. Therefore, functional gastrointestinal disorders, although seemingly benign conditions, can lead to serious long-term complications and should not be overlooked.

## Consent

The patient provided informed consent for publication of his clinical details.
