# Supplementary material for: Functional abdominal pain causing Scurvy, Pellagra, and Hypovitaminosis A
Source: F1000Res. 2014 Feb 4;3:35. [Version 1] doi: 10.12688/f1000research.3-35.v1 (PMC3976101; doi:10.12688/f1000research.3-35.v1)
Supplement: Peer review history

# Peer review: Referee response for version 1

**Published:** 2014-03-28

**Authors:** Sarah Sheibani

**Affiliations:** 1 Department of Clinical Medicine, Keck School of Medicine, University of Southern California, Los Angeles, CA, USA

This is a very well-written and informative case report that highlights a potential complication that can occur in an apparently benign disorder. Functional abdominal pain is one of the most common disorders evaluated by gastroenterologists. To my knowledge, this is the first report to suggest that vitamin deficiencies should be checked in patients with functional abdominal pain that present with malnutrition and diarrhea along with unexplained clinical findings, such as a rash.

I have read this submission. I believe that I have an appropriate level of expertise to confirm that it is of an acceptable scientific standard.

# Peer review: Referee response for version 1

**Published:** 2014-02-05

**Authors:** Bruce Bistrian

**Affiliations:** 1 Nutrition Infection Laboratory, Department of Medicine, Beth Israel Deaconess Medical Center, Boston, MA, USA

This is an interesting case report that demonstrated the appearance of undetectable vitamin A and C levels in the setting of dermatologic evidence of niacin deficiency (pellagra) and vitamin C (scurvy) that developed in an individual with severe gastrointestinal symptoms but without weight loss presumed secondary to a functional disorder.   

That severe dietary deficiencies could develop over the period of years from a disturbed diet is likely, and the laboratory evidence for deficiency is not refutable for vitamins A and C. However it would be interesting to know whether there was biochemical evidence for niacin deficiency and whether the presumed pellagrous skin lesions responded to vitamin supplementation, since the routine B vitamin fortification of grain products in developed countries would make this somewhat less likely. Although the authors state that red meat was tolerated to some extent, it is not likely that it would be a substantial component of the diet, since meat is a good source of essential nutrients. The publication of this report is important to remind us that nutritional deficiencies can develop under such conditions.

I have read this submission. I believe that I have an appropriate level of expertise to confirm that it is of an acceptable scientific standard.
